# Study motivations, specialty preferences, and empathy of dental students in a Nigerian university

**DOI:** 10.11604/pamj.2022.41.328.33123

**Published:** 2022-04-22

**Authors:** Priscilla Okhiabigie Ameh, Omolara Gbonjubola Uti, Opeyemi Oluwayemisi Daramola

**Affiliations:** 1 Department of Preventive Dentistry, Faculty of Dental Sciences, College of Health Sciences, University of Jos, Plateau State, Nigeria,; 2 Department of Preventive Dentistry, Faculty of Dental Sciences, College of Medicine, University of Lagos, Lagos State, Nigeria,; 3 Department of Preventive Dentistry, Lagos University Teaching Hospital, Lagos, Lagos State, Nigeria

**Keywords:** Dental, students, empathy, professional, motivation, Nigeria

## Abstract

**Introduction:**

dental training aims to produce committed dentists who are professional and empathetic in providing patient-centered oral healthcare and improved quality of life. This study aimed to assess the motives of dental students to study dentistry, their specialty preferences, and their empathy scores by motives and specialty preference.

**Methods:**

this cross-sectional study utilized a self-administered questionnaire designed to assess the empathy measured by the Jefferson Scale of Empathy-Health Professions Student version, motives to study dentistry, dental specialty preferences among students of a dental school in Nigeria. Differences in mean empathy across gender, motives to study dentistry, and specialty preferences were analysed with t-test and ANOVA. P-values of <0.05 were considered significant.

**Results:**

a total of 211 students participated fully in the study with a mean age of 21.19 years ± 2.43 and 140 (66.4%) females. Personal interest (27.0%) and failure of admission to other undergraduate programmes (20.9%) were the top two motives for studying dentistry. Oral and maxillofacial surgery (OMFS) was the most preferred specialty (44.1%) and community dentistry and periodontology were the least preferred (2.8%) respectively. The total mean empathy score was 104.06 ± 19.1. The highest empathy score for motivation was for high income from dentistry (112.33 ± 13.34). The participants who preferred paediatric dentistry had the highest empathy score (108.73 ± 13.68).

**Conclusion:**

knowledge of the empathy levels, the motivation for studying dentistry, and preferences for particular specialties may encourage trainers to ensure the development of a positive attitude among dental students that is professional and empathetic.

## Introduction

Dentistry is a highly skilled profession that involves years of intense and demanding undergraduate and postgraduate specialist training. Thus, it requires that both undergraduate and postgraduate students, as well as practicing dentists be committed and dedicated to providing quality patient care. Identifying the motives which drive the decision to study dentistry is important. This helps in understanding the psychological makeup and professional expectations of the students as well as the potential job satisfaction as dentists [1]. Several factors contribute to the decision to study dentistry as a future career goal. Gender, expected income, family influence, personal interest, a desire to help people improve their oral health, etc. may singly or together motivate this choice [1-3].

The different dental specialties aim to provide holistic and specialized oral healthcare to the community. However, there is an uneven distribution of these specialties among dentists in Nigeria [4-6]. Oral and maxillofacial surgery (OMFS) has proved to be a major attraction among undergraduate students and dentists who have expressed a desire towards postgraduate specialization [4-6]. Other specialties have not fared so well, leading to a cumulative shortage of specialists in these areas [4,5]. Empathy has been described in the context of patient care as “an understanding (rather than a feeling) of the patient´s pain, experiences, concerns and perspectives, the capability to communicate this understanding to the patient and an intention to help” [7,8]. High empathetic orientation in physicians impacts positively on the development of optimum doctor-patient communication, the accuracy of diagnosis, compliance, and ultimately patient satisfaction and improved health [9-11]. Medical specialties focused on providing primary care and requiring sustained interactions with patients appear to be more attractive to medical students and physicians with higher empathetic traits [10,12,13]. It is unclear if the empathetic orientation of dental students plays a role in the preference for particular dental specialties.

The information on the motives and specialty preferences of undergraduate and practicing dentists in Nigeria can be further enriched [1,5,6,14]. The data on the assessment of empathy in the context of study motives and specialty preferences among both dental students and practicing dentists are scarce. The purpose of this study is to identify the motives for choosing to study dentistry, the specialty preferences and, the related empathy levels of undergraduate dental students of the University of Lagos, Nigeria.

## Methods

**Study setting:** this study was conducted in the University of Lagos, which is one of two government-owned universities in Lagos State, Nigeria with a dental school. Lagos State is situated in the South-West of Nigeria in West Africa.

**Study design, population, and sampling:** this was a cross-sectional study that utilized a self-administered questionnaire to acquire information from the first to sixth year dental students. The study population consisted of undergraduate dental students of the Faculty of Dentistry, College of Medicine, University of Lagos, Lagos, Nigeria from December 15^th^, 2015 to January 12^th^, 2016. All students in the first to sixth year were eligible for the study. Permission to use the Jefferson Scale of Empathy-Health Professions Student (JSE-HPS) version for this study, allowed for only a maximum of 250 participants. Participants were sampled using a proportion to size method based on the number of students in each year of study as described in a previous publication [15]. Systematic sampling was carried out using a determined sampling interval of the class lists to select 234 students from the six years of study.

**Study instrument and data collection:** a self-administered questionnaire consisting of four sections was utilized to collect information from the subjects. Section A obtained information on the demographic variables such as age, gender and, year of study. Section B of the questionnaire was used to obtain information on the motivations to study dentistry as an undergraduate course and required a “yes” or “no” response, allowing for multiple responses. Section C was to obtain information on their specialty preferences from a list of nine dental specialties. Section D of the questionnaire was the structured JSE-HPS, which was obtained from the Center for Research in Medical Education and Health Care, Thomas Jefferson University, Pennsylvania, USA. It is a 20-item 7-point Likert scale (strongly disagree = 1, strongly agree = 7), with an individual score range of 20 to 140. The scoring system has been described previously [15,16]. The JSE-HPS measures the empathy levels of healthcare professional students in the context of patient care. Individual JSE scores are directly proportional to the empathy levels. Thus, higher JSE scores demonstrate a tendency towards positive empathic behaviour in patient care [16]. The questionnaires were distributed among the participants in their lecture rooms towards the end of their academic year. The filled questionnaires were collected immediately.

**Data analysis:** data were analysed with the IBM SPSS version 23.0.0.0 (IBM Inc., Chicago, IL). Incomplete entries were excluded from the analysis. Information on the year of study, age, gender, motives to study dentistry, specialty preferences, and the JSE-HPS to assess empathy were the variables analysed. Categorical variables such as gender, year of study, marital status, motives for studying dentistry, and specialty preference were summarized as frequencies and proportions. Continuous variables such as age and JSE scores were expressed as means and standard deviations. A chi-square test was used to determine any association between gender and motives to study dentistry. Independent t-test and analysis of variance (ANOVA) were used to analyse differences in mean empathy across gender, motives to study dentistry, and specialty preferences. P-values of <0.05 were considered significant.

**Ethical consideration:** the study protocol was approved by the Lagos University Teaching Hospital Health Research Ethics Committee (ADM/DCST/HREC/APP/573). Permission to use the JSE-HPS version was obtained from the Center for Research in Medical Education and Health Care, Jefferson Medical College of Thomas Jefferson University, Pennsylvania, USA. Participants´ consent was also obtained.

## Results

**Sociodemographic characteristics of the participants:** Table 1 displays the sociodemographic characteristics of the participants. A total of 211 students filled all the sections of the questionnaire. The mean age of the participants was 21.19 years ± 2.43. More than half (140, 66.4%) of them were females.

**Table 1:** characteristics of participants (n = 211)

Variables	Frequency (%)
**Sex**	
Male	71 (33.6)
Female	140 (66.4)
**Year of study**	
First year	28 (13.3)
Second year	35 (16.6)
Third year	29 (13.7)
Fourth year	46 (21.8)
Fifth year	41 (19.4)
Sixth year	32 (15.2)
**Marital status**	
Single	206 (97.6)
Married	5 (2.4)
**Religion**	
Christianity	168 (80.0)
Islam	40 (19.0)
Others	2 (1.0)
**Previous university degree**	
Yes	1 (0.5)
No	210 (99.5)
**Close relation is a dentist**	
Yes	21 (9.9)
No	190 (90.1)

**Figure 1:**
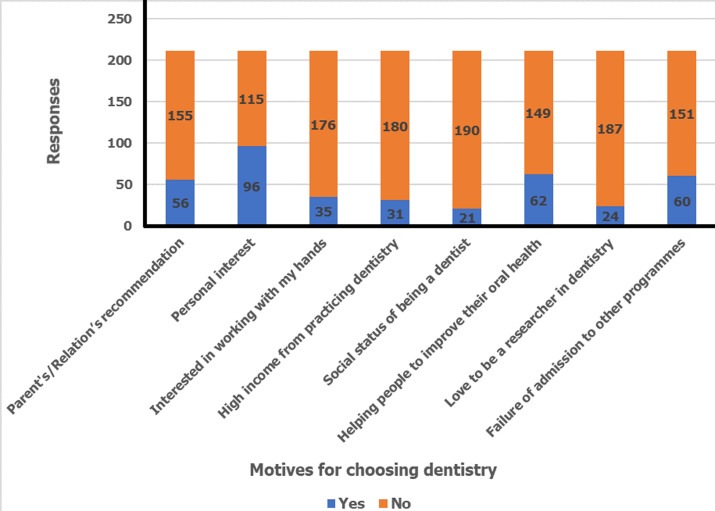
motivations for choosing to study dentistry

**Motivations for studying dentistry:** the motives for choosing to study dentistry are illustrated in Figure 1. Personal interest in dentistry (96, 45.5%) was the topmost motive to study dentistry. To help people improve their oral health ranked second (62, 24.4%). The social status of being a dentist was the least common motive (21, 10.0%) and only 8 (5.7%) of the females selected it. Table 2 shows the association between gender and the motives to study dentistry. There was a statistically significant association between gender and social status as a motive to study dentistry (χ^2^= 8.339, df = 1, P = 0.004).

**Table 2:** association between gender and motives for choosing to study dentistry (n = 211)

Motivation	Responses*	Male (%)	Female (%)	Total (%)	X^2^	P value
Parent's/relation's recommendation	Yes	21 (37.5)	35 (62.5)	56 (100.0)	0.506	0.477
	No	50 (32.3)	105 (67.7)	155 (100.0)		
Personal interest	Yes	34 (35.4)	62 (64.6)	96 (100.0)	0.246	0.620
	No	37 (32.2)	78 (67.8)	115 (100.0)		
Interested in working with my hands	Yes	10 (28.6)	25 (71.4)	35 (100.0)	0.485	0.486
	No	61 (34.7)	115 (65.3)	176 (100.0)		
High income from practicing dentistry	Yes	13 (41.9)	18 (58.1)	31 (100.0)	1.118	0.290
	No	58 (32.2)	122 (67.8)	180 (100.0)		
Social status of being a dentist	Yes	13 (61.9)	8 (38.1)	21 (100.0)	8.339	0.004
	No	58 (30.5)	132 (69.5)	190 (100.0)		
Helping people improve their oral health	Yes	24 (38.7)	38 (61.3)	62 (100.0)	1.007	0.316
	No	47 (31.5)	102 (68.5)	149 (100.0)		
I would love to be a researcher in dentistry	Yes	10 (41.7)	14 (58.3)	24 (100.0)	0.780	0.377
	No	61 (32.6)	126 (67.4)	187 (100.0)		
Failure of admission to other undergraduate programmes	Yes	19 (31.7)	41 (68.3)	60 (100.0)	0.148	0.701
	No	52 (34.4)	99 (65.6)	152 (100.0)		

* Multiple responses

**Figure 2:**
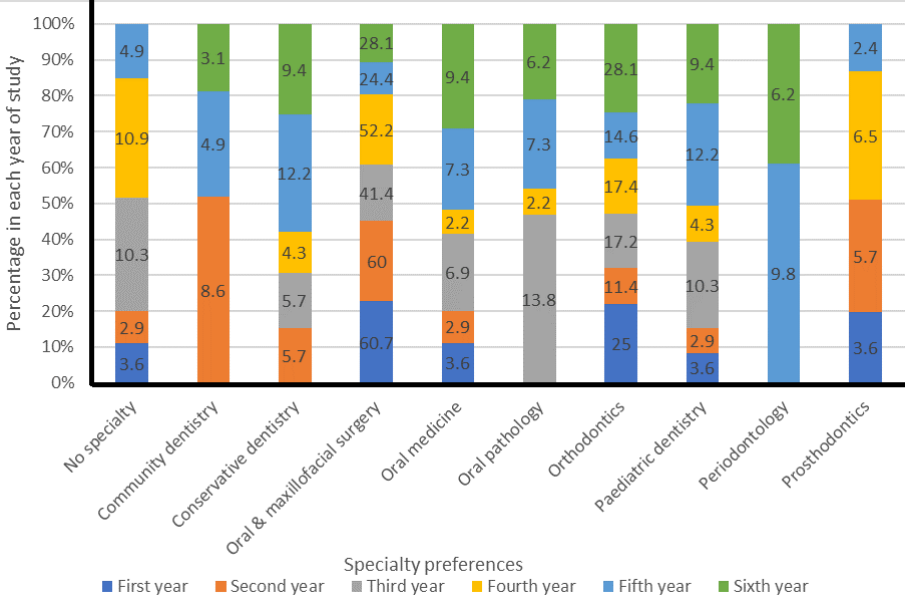
specialty preferences of participants across the six years of study (n = 211)

**Specialty preferences of participants:** overall, 199 (94.3%) students indicated a specialty preference. Oral and maxillofacial surgery (93,44.1%) was the most preferred dental specialty overall. The second most preferred specialty was orthodontics (39, 18.5%). The least preferred specialties were community dentistry and periodontology, in which only 6 (2.8%) of the students respectively, expressed an interest. The female to male ratio for preference for paediatric dentistry was 14: 1 (Table 3). The majority of the participants in every year of study (41.4% - 60.7%) preferred OMFS. Figure 2 illustrates that all students in the sixth year had a specialty preference. A preference for periodontology was indicated by participants in the fifth and sixth years only.

**Table 3:** specialty preferences and gender of participants (n = 211)

Specialty preference	Male (%)	Female (%)	Total (%)
No specialty	2 (0.9)	10 (4.7)	12 (5.7)
Community dentistry	1 (0.5)	5 (2.4)	6 (2.8)
Conservative dentistry	4 (1.9)	8 (3.8)	12 (5.7)
Oral and maxillofacial dentistry	42 (19.9)	51 (24.2)	93 (44.1)
Oral medicine	4 (1.9)	7 (3.3)	11 (5.2)
Oral pathology	5 (2.4)	5 (2.4)	10 (4.7)
Orthodontics	7 (3.3)	32 (15.2)	39 (18.5)
Paediatric dentistry	1 (0.5)	14 (6.6)	15 (7.1)
Periodontology	3 (1.4)	3 (1.4)	6 (2.8)
Prosthodontics	2 (0.9)	5 (2.4)	7 (3.3)
Total	71 (33.6)	140 (66.6)	211 (100.0)

**Empathy levels, motives for studying dentistry, and specialty preferences:** Table 4 shows the empathy scores and the differences in the mean scores for both study motives and specialty preferences. The total mean empathy score was 104.06 ± 19.17. The mean empathy score was higher for the female students (105.25 ± 17.23) than for males (101.68 ± 31) although, this was not statistically significant (t = 1.287, df = 1, P = 0.200). In terms of motive to study dentistry, students motivated by high income had the highest empathy score of 112.33 ± 13.34. Regarding specialty preferences, students who preferred paediatric dentistry had the highest empathy level at 108.73 ± 13.68. There were no statistically significant differences in the mean empathy levels due to motivation (F =0.763, df=9, P=0.651) or, due to specialty preferences (F = 0.306, df = 9, P = 0.972).

**Table 4:** mean empathy scores of participants (n = 211)

Variables	Mean empathy scores	SD	95% CI	P value
**Sex**				
Male	101.68	22.31	96.39 - 106.96	0.200*
Female	105.26	17.32	102.37 - 108.16	
**Motives for choosing dentistry**				
No reason	95.00	.	.	0.651†
Immediate family member is a dentist	105.27	12.57	98.30 - 112.23	
Failure to be admitted to other undergraduate programmes	107.41	21.69	100.81 - 114.00	
Parents'/relations' recommendation	103.90	14.02	98.57 - 109.23	
Personal interest in dentistry	101.26	20.27	95.89 - 106.64	
Interest in working with my hands	103.83	22.84	89.32 - 118.34	
High income from dentistry	112.33	13.34	102.08 - 122.59	
Social status of being a dentist	107.40	4.39	101.95 - 112.85	
Helping people improve their oral health	99.39	23.14	90.42 - 108.37	
Love being a researcher	108.55	14.05	99.11 - 117.98	
**Specialty preference**				
No specialty	107.75	8.99	102.03 - 113.47	0.972†
Community dentistry	98.67	30.66	66.49 - 130.84	
Conservative dentistry	105.25	28.71	87.01 - 123.49	
Oral and maxillofacial dentistry	103.81	18.38	100.02 - 107.59	
Oral medicine	103.55	15.87	92.88 - 114.21	
Oral pathology	107.10	13.34	97.56 - 116.64	
Orthodontics	101.92	24.20	94.08 - 109.77	
Paediatric dentistry	108.73	13.68	101.16 - 116.31	
Periodontology	100.50	10.52	89.46 - 111.54	
Prosthodontics	105.00	11.86	94.03 - 115.97	
Total	104.06	19.17	101.46 - 106.66	

SD: standard deviation: CI: confidence interval; * t test; † ANOVA

## Discussion

There were more females than males studying dentistry in this population. The higher number of female students studying dentistry has also been reported in another study among Nigerian dental students some years ago [2]. Conversely, another study among Nigerian final-year dental students had more males [1]. Over the past 50 years, there has been an increase in the number of women studying and practicing dentistry [14,17-19]. Flexible work hours and easier work-life balance during practice have been reported as the two major motivations among females who study dentistry [19]. However, in this study, the highest motivation among the females was personal interest, followed by helping people improve their health. In both sexes, personal interest was the most popular motive to study dentistry. A previous study among clinical dental students in Nigeria had also reported interest as the highest motivating factor [2]. In contrast, another study among final-year dental students in Nigeria reported motivation from parents as the top motive to study dentistry [1]. Two types of interest have been described in educational research: 1); The situational interest that is a psychological state that involves improved attention, effort, and affects, and is experienced in a particular moment and; 2), the individual interest, that is an enduring preference to reengage with a specific object or topic over time [20]. Individual interest has been shown to have a positive impact on academic motivation [21]. This augur well for continual learning and commitment in both the study and practice of dentistry. This is very essential in the case of Nigeria, where there exists a severe shortage of dentists with an attendant brain drain [22]. These both result in a reduced number of trained dental professionals and a deterioration of the oral health care service delivery [22,23]. Failure to obtain admission into other courses was the second most popular motive to study dentistry. It could be inferred from this, that the desire to study dentistry might not be a priority in this group of students and thus may diminish the likelihood to practice dentistry or pursue specialist training. This would further worsen the delivery of oral healthcare services in Nigeria [22].

Social status was seen to have the least influence in studying dentistry in this study. Prestige, which can be akin to social status was reported as the second highest motivating factor in a study among clinical dental students in Nigeria [2]. In another study, prestige was shown to have little influence on specialty choice among final-year dental students [5]. Consideration of prestige is an increasingly important influential trend among practicing dentists in their choice of specialty training [14]. Thus, the low influence of social status among the students in this study might increase after graduation, when they begin practicing and become more aware of the societal high status and earning capacity that ensues from being a dentist.

A desire to help people which is an altruistic trait was not a priority in this population. A similar finding has also been reported in other studies in which altruism was not a major factor influencing the students´ choice of dentistry rather, professional status, financial rewards, and security were stronger motives [3,24]. Altruism involves actions that are for the good and self-interest of another, at times at a cost or risk to oneself. This is fundamental to healthcare practice and is embodied in the origins of the hippocratic oath [25,26]. The apparent dichotomy between helping people improve their oral health, a seemingly altruistic motive, and the low empathy level is perplexing. In recent years, there has been a growing concern about the decline of altruism in the medical practice and this could be detrimental to the recruitment and retention of healthcare professionals as well as workforce planning [26].

Oral and maxillofacial surgery was shown to be the most popularly preferred specialty in this study. This corroborates previous reports among both undergraduate students and practicing dentists [4-6]. Possible reasons might include a general basic understanding of a dentist to be one who surgically removes painful teeth and an early introduction to OMFS in clinical training. A significant number of those who expressed a preference for paediatric dentistry were females. This is in contrast with a study among final-year dental students, where the majority of those who indicated an interest in paediatric dentistry were males [1]. There were very low preferences for prosthodontics, community dentistry, and periodontology. Prosthodontics and periodontology have previously been observed to be unpopular specialties among both undergraduate students and practicing dentists [4-6]. Community dentistry has also been shown as a not very popular specialty preference [4,5]. The effect of a low preference for other dental specialties means fewer dentists specialize in these areas. There is thus both a shortage of specialists to provide the necessary oral healthcare and the trainers for undergraduate and postgraduate dental students in these specialties.

Ironically in this study, students whose motive to study dentistry was helping people improve their oral health had the second to the least mean empathy score. It is expected that this altruistic motive would manifest with a higher empathetic orientation. Disconcertingly, high income which may be regarded as a pecuniary motive had the highest empathy score. In contrast, it has been shown that humanistic, positive attitudes towards doctor-patient relationships and altruistic motivations have a positive relationship with empathy among students of other healthcare professions [27,28].

The higher empathetic orientation in the students who prefer paediatric dentistry may be due to the proportionally higher number of females among them. Behaviour management of the child dental patient aims to establish effective communication, alleviate fear and anxiety and develop a trusting relationship with the patient that will ultimately allow a positive experience for both dentist and patient, ensure the provision of quality dental care and promote a positive attitude towards dental care and oral health in the child [29]. A high degree of empathy would thus be required in managing the child dental patient to achieve this goal.

**Limitations:** some of the limitations of this study include its single institutional sampling which constraints the generalization of the findings. Another is the use of a self-administered questionnaire. Although this is quick and practical, there is the tendency for obtaining socially acceptable responses which may be invalid [30]. Further studies are required in exploring the role of empathy and its influence on study motives and specialty preferences among dental students in Nigeria.

## Conclusion

Knowledge of the empathy levels, the motivation for studying dentistry, and preferences for particular specialties among dental students may guide the admission process and the development of a curriculum that enriches the learning experience, enhances interpersonal skills, and encourage the development of a positive attitude among dental students, that is professional and empathetic and promotes patient-centred oral healthcare delivery.

### What is known about this topic

- The motives for studying dentistry are complex and dynamic;
- Oral and maxillofacial surgery is the most common specialty preference among dental students;
- Empathy is crucial to a patient-centered oral healthcare delivery.

### What this study adds

- Altruistic motivations and thus empathy seems to have little influence on choosing to study dentistry;
- Empathetic orientation is higher for those who prefer paediatric dentistry;
- The role of empathy in study motivations and specialty preferences among Nigerian dental students need further exploration.
